# Supplementary material for: Cervicovaginal Microbiome Composition Is Associated with Metabolic Profiles in Healthy Pregnancy
Source: mBio. 2020 Aug 25;11(4):e01851-20. doi: 10.1128/mBio.01851-20 (PMC7448280; doi:10.1128/mBio.01851-20)
Supplement: FIG S1 [file mBio.01851-20-sf001.pdf]

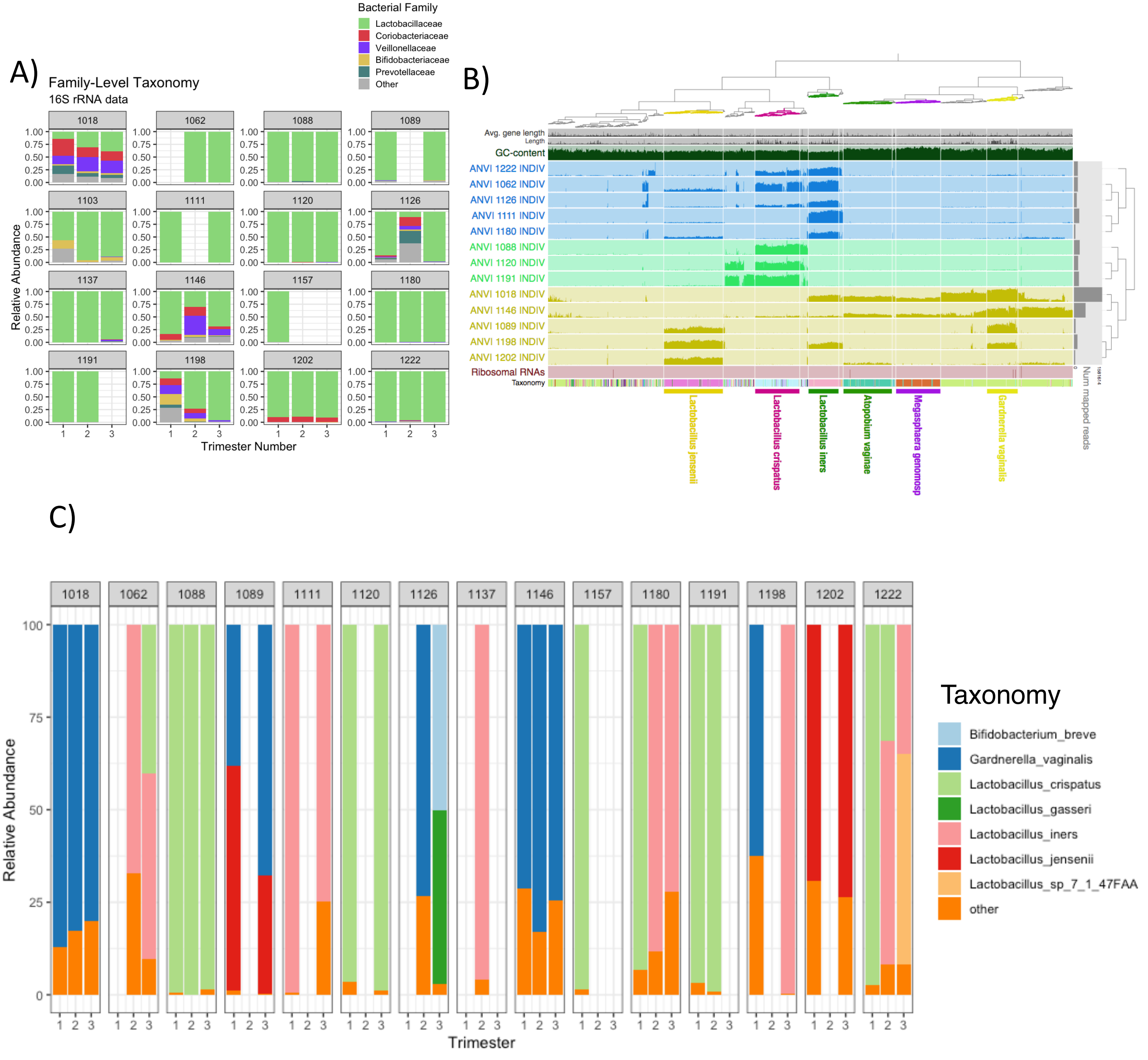

Supp. Fig. 1: A) Stacked bar plots of 16S rRNA gene taxonomy, collapsed down to the family level. ”Other” category contains taxa at a mean relative abundance of 1% or less. B) Anvi’o plot illustrating the abundance of contigs (>= 2,000bp) for each subject (trimesters combined). The participants are colored according to the most abundant microbe present (*L. iners* (blue), *L. crispatus* (green), *G. vaginalis* (yellow)). Short read taxonomic assigner Kaiju was used to assign taxonomy to the contigs. C) Species level taxonomy of 35 shotgun metagenomic samples, using the MiDAS taxonomic classifier.
