# Supplementary material for: Cervicovaginal Microbiome Composition Is Associated with Metabolic Profiles in Healthy Pregnancy
Source: mBio. 2020 Aug 25;11(4):e01851-20. doi: 10.1128/mBio.01851-20 (PMC7448280; doi:10.1128/mBio.01851-20)
Supplement: FIG S2 [file mBio.01851-20-sf002.pdf]

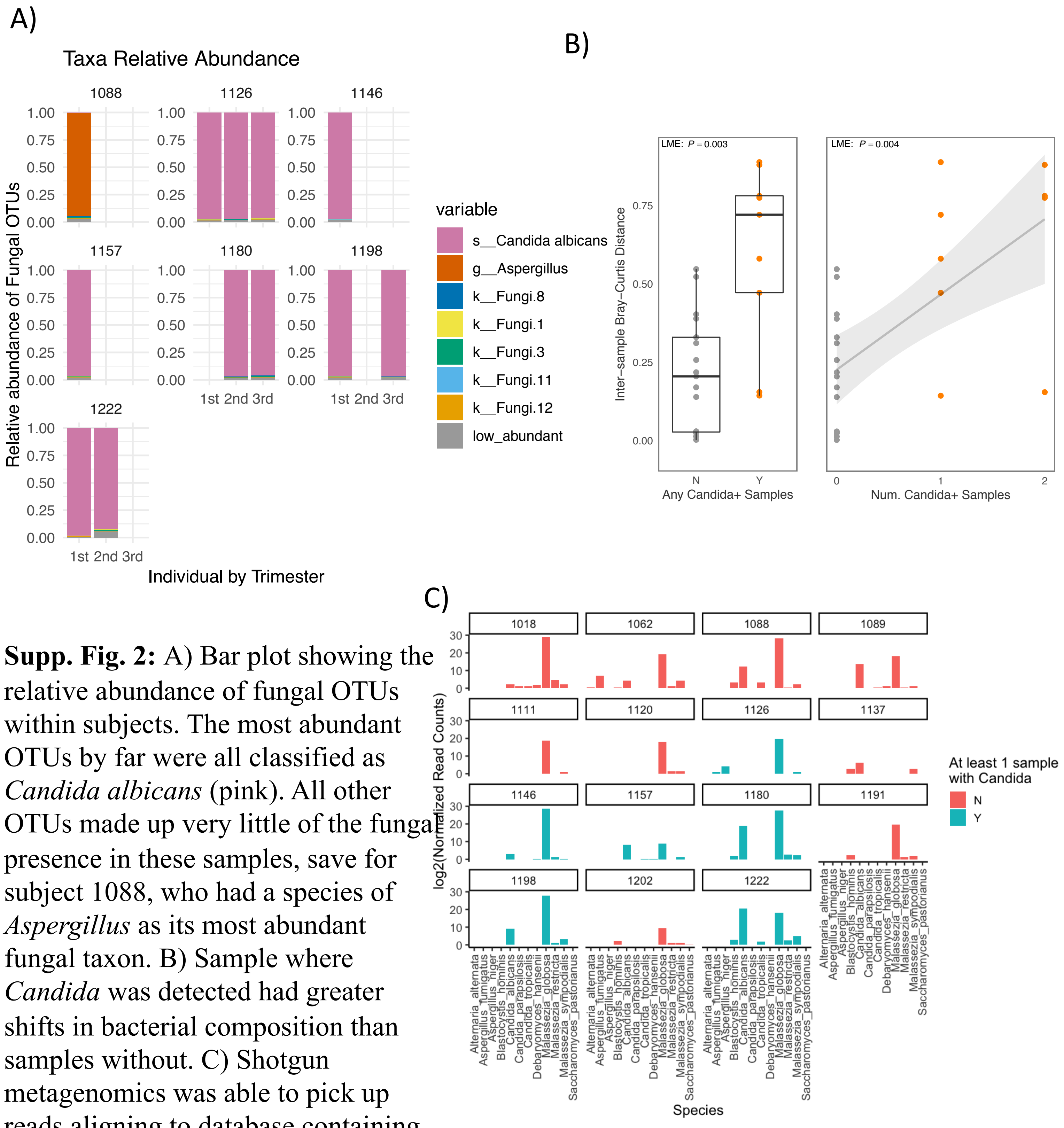

Supp. Fig. 2: A) Bar plot showing the relative abundance of fungal OTUs within subjects. The most abundant OTUs by far were all classified as *Candida albicans* (pink). All other OTUs made up very little of the fungal presence in these samples, save for subject 1088, who had a species of *Aspergillus* as its most abundant fungal taxon. B) Sample where *Candida* was detected had greater shifts in bacterial composition than samples without. C) Shotgun metagenomics was able to pick up reads aligning to database containing human-associated fungal species and confirmed ITS results and classified additional taxa.
