# Supplementary material for: Cervicovaginal Microbiome Composition Is Associated with Metabolic Profiles in Healthy Pregnancy
Source: mBio. 2020 Aug 25;11(4):e01851-20. doi: 10.1128/mBio.01851-20 (PMC7448280; doi:10.1128/mBio.01851-20)
Supplement: FIG S3 [file mBio.01851-20-sf003.pdf]

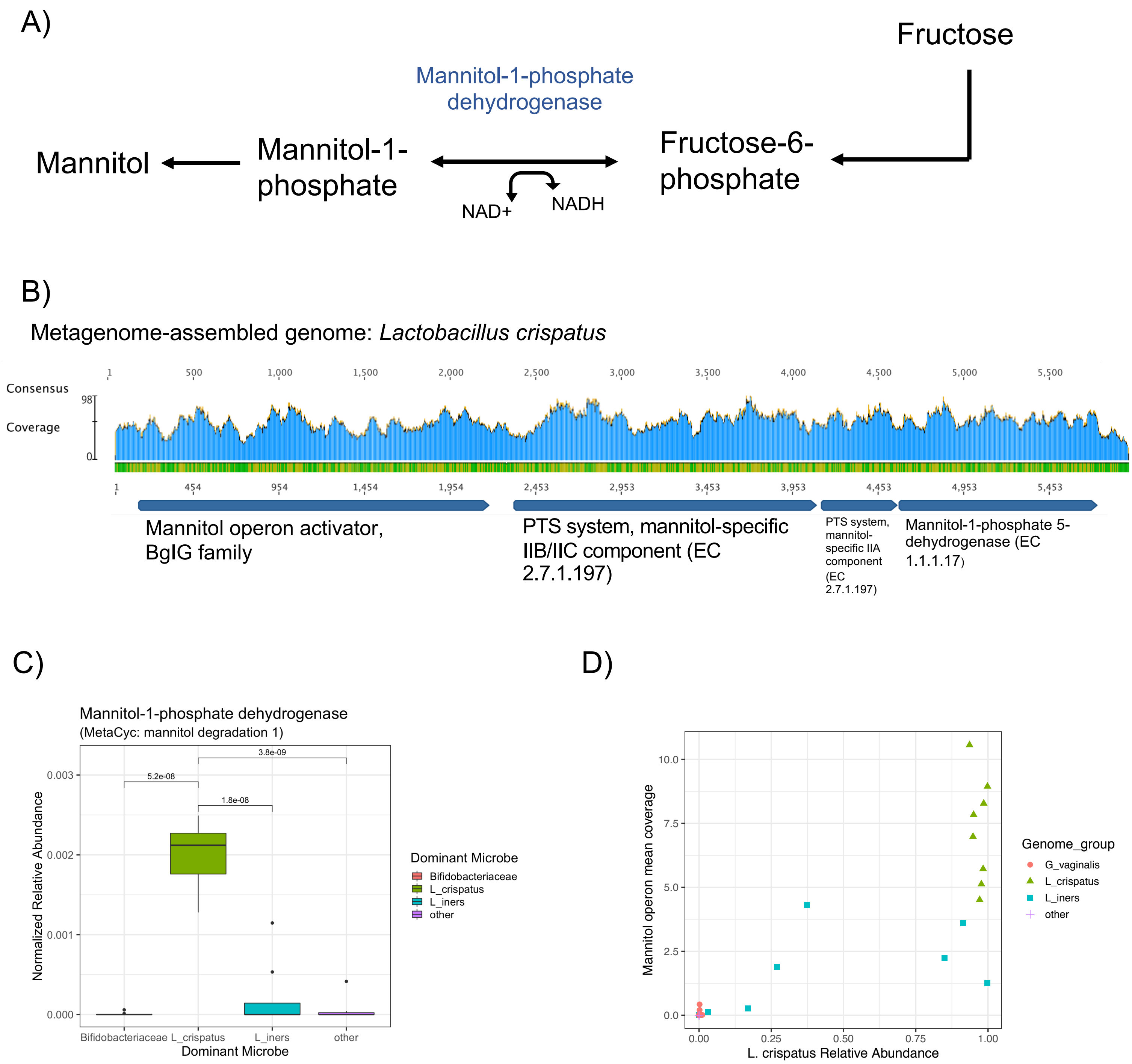


**Supp. Fig. 3:** A) Schematic of mannitol production. B) Coverage the mannitol operon from the metagenomes of individuals that had high abundances of *L. crispatus* in their samples. The operon was assembled using taxonomically assigned contigs in Anvio v5 and annotated using Patric. C) Analysis of the HUMAnN2 output, specifically the mannitol degradation pathway. Pairwise T-tests of *L. crispatus* against other dominant microbes. D) A Relationship between mean coverage of that operon with relative abundance of *L. crispatus*.
