# Supplementary material for: Cervicovaginal Microbiome Composition Is Associated with Metabolic Profiles in Healthy Pregnancy
Source: mBio. 2020 Aug 25;11(4):e01851-20. doi: 10.1128/mBio.01851-20 (PMC7448280; doi:10.1128/mBio.01851-20)
Supplement: FIG S4 [file mBio.01851-20-sf004.pdf]

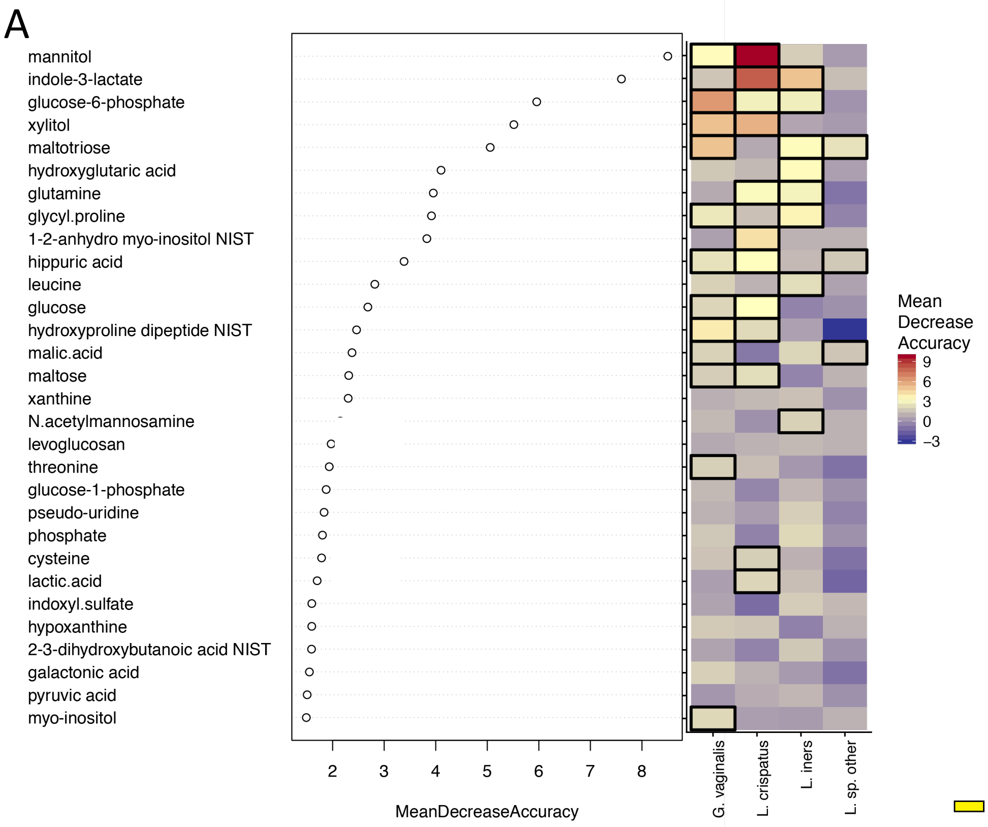

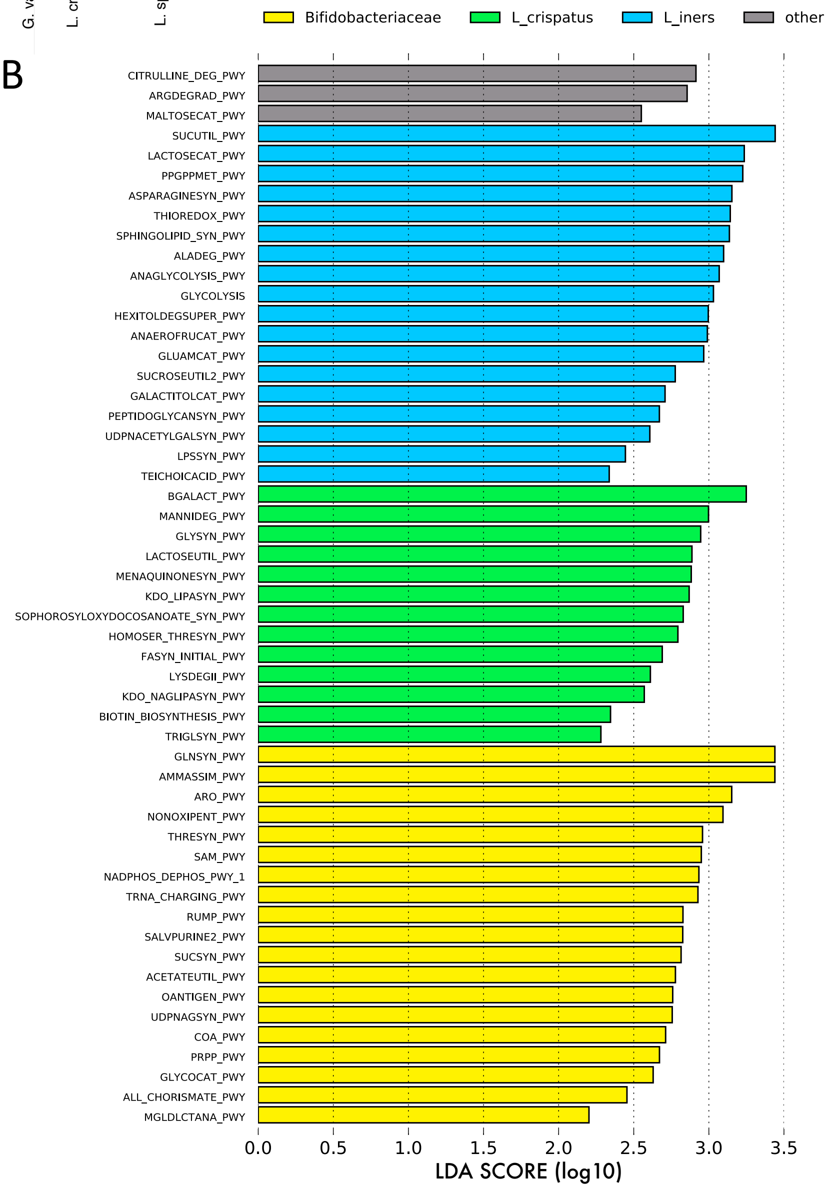

Supp. Fig. 4: A) Permutated random forest recapitulates the metabolites that drive differences between the vaginal communities. The heatmap shows the mean decrease in accuracy associated with the specific microbiomes. Bold boxes around the heatmap cell indicates statistical significance of that feature at $p < 0.05$. B) Lefse analysis of annotated HUMAnN2 output comparing the enrichment of functional pathways between vaginal microbiomes with abundant *Bifidobacteriaceae (G. vaginalis), L. crispatus, L. iners,* or other. This analysis was done using the -no_stratify flag for humann2, which analyzes the data without the taxa specific information.
