# Supplementary material for: Cervicovaginal Microbiome Composition Is Associated with Metabolic Profiles in Healthy Pregnancy
Source: mBio. 2020 Aug 25;11(4):e01851-20. doi: 10.1128/mBio.01851-20 (PMC7448280; doi:10.1128/mBio.01851-20)
Supplement: FIG S5 [file mBio.01851-20-sf005.pdf]

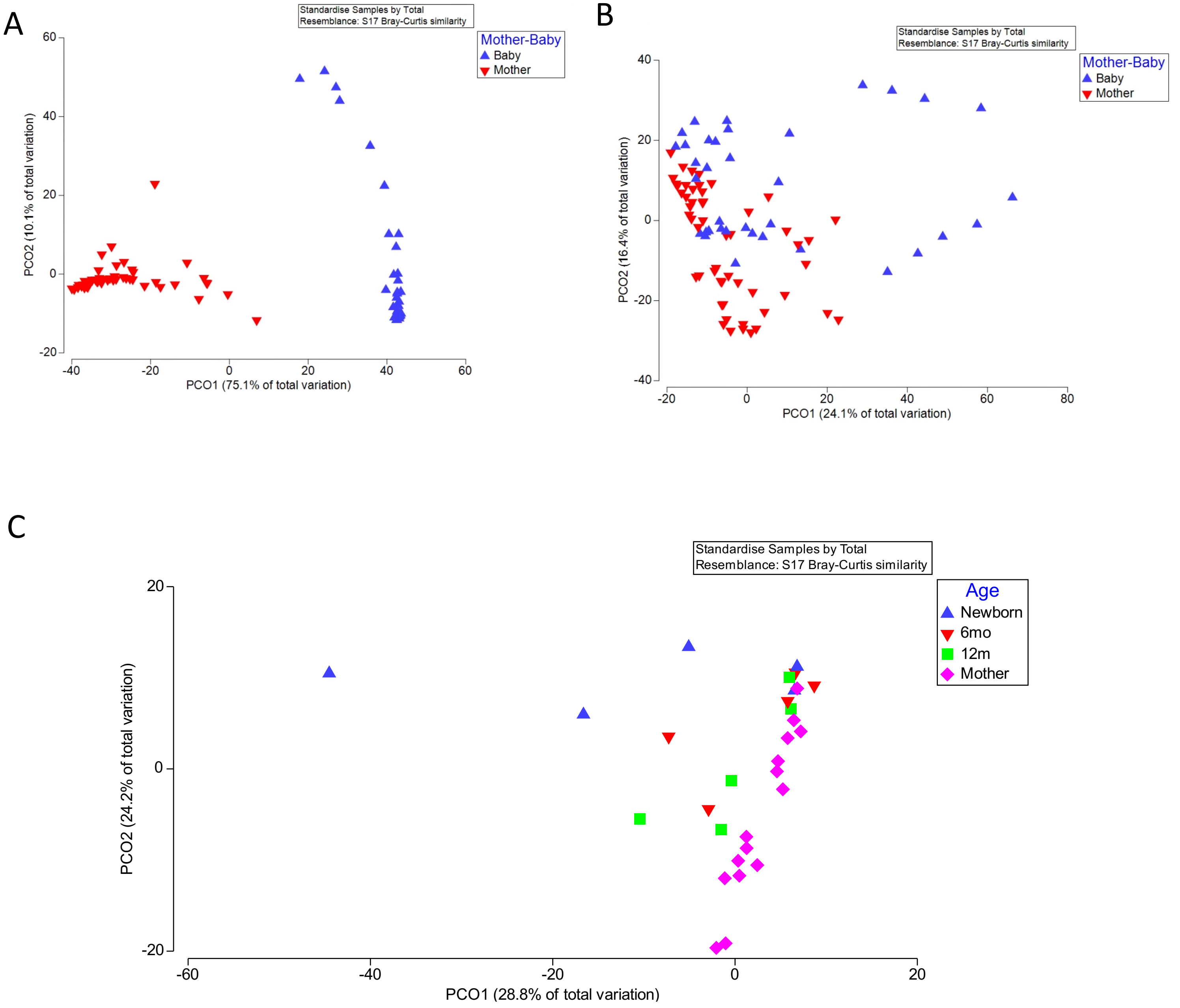

Supp. Fig. 5: A) Principle coordinates ordination of saliva LC-MS/MS lipidomics, colored by whether the sample originated from mother or child. B) Principle coordinates ordination of saliva metabolomes by GC-TOF, colored by whether the sample originated from mother or child. C) Principle coordinates ordination of urine lipidomes, colored by age. All timepoints for mothers (n=15 samples) were used (i.e. Trimesters 1-3).
