# Supplementary material for: Cervicovaginal Microbiome Composition Is Associated with Metabolic Profiles in Healthy Pregnancy
Source: mBio. 2020 Aug 25;11(4):e01851-20. doi: 10.1128/mBio.01851-20 (PMC7448280; doi:10.1128/mBio.01851-20)
Supplement: FIG S6 [file mBio.01851-20-sf006.pdf]

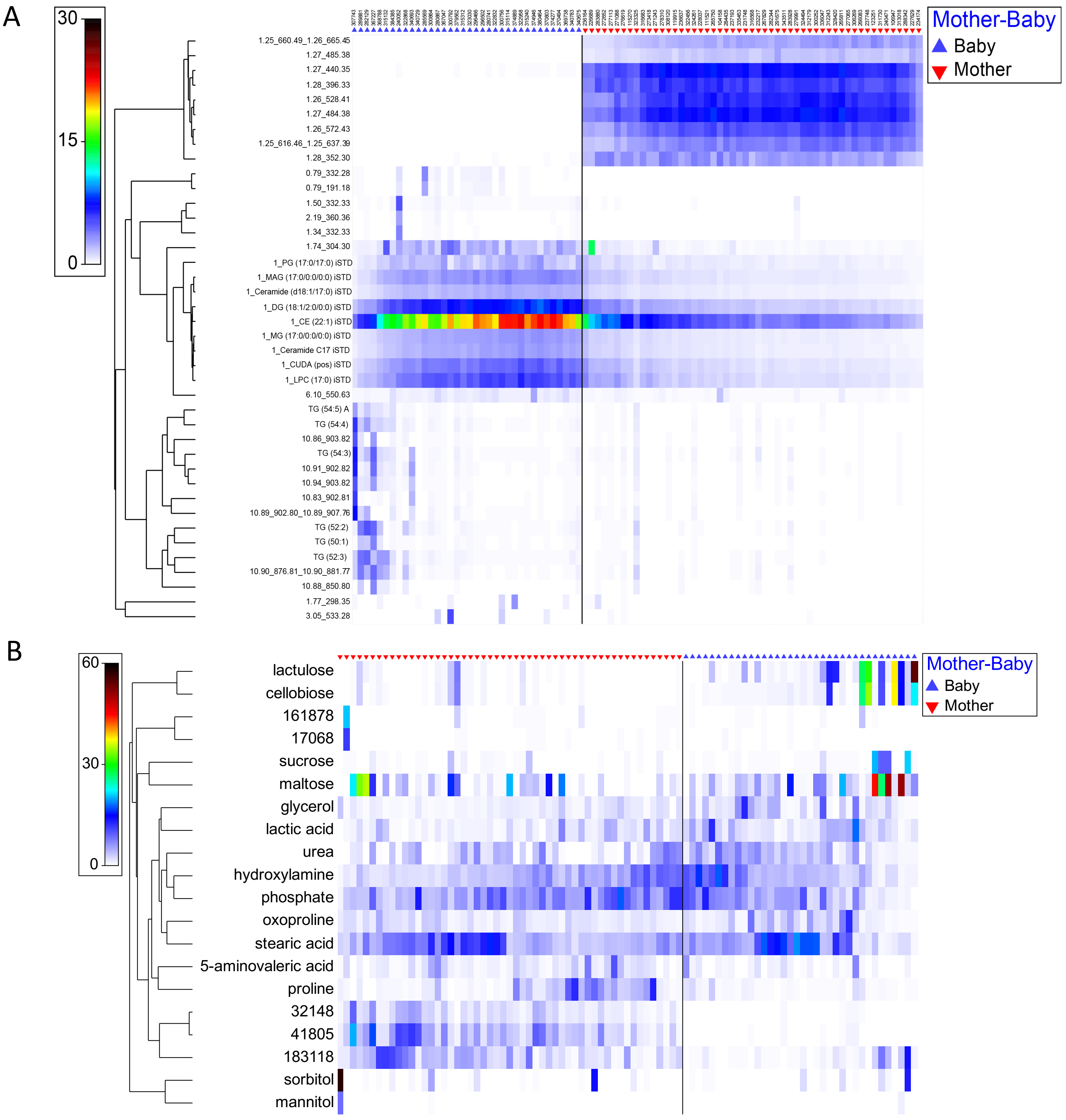

Supp. Fig. 6: A) Analysis of the top 50 lipidomic metabolites and B) 20 top GC-TOF metabolites from saliva that had the highest mean abundance across all samples using Primer-e software. Metabolites were initially standardized within each sample, and then across metabolites. Color indicates this normalized abundance of the metabolite. When present, annotations were used for data.
