# Supplementary material for: Cervicovaginal Microbiome Composition Is Associated with Metabolic Profiles in Healthy Pregnancy
Source: mBio. 2020 Aug 25;11(4):e01851-20. doi: 10.1128/mBio.01851-20 (PMC7448280; doi:10.1128/mBio.01851-20)
Supplement: FIG S7 [file mBio.01851-20-sf007.pdf]

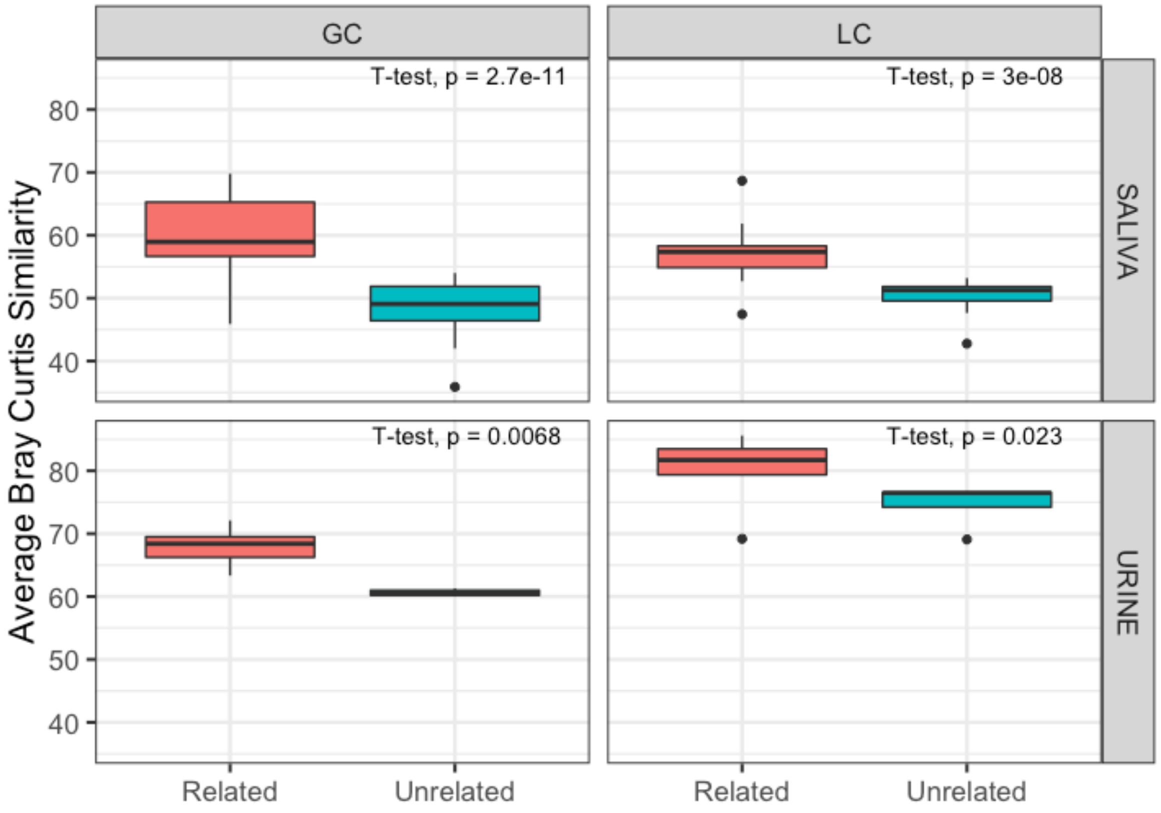

Supp. Fig. 7: Similarity of urine and saliva metabolomes between related and unrelated individuals. Related mothers and children have significantly more similar saliva and urine metabolomes than unrelated individuals. Graph shows average bray Curtis similarity between related and unrelated individuals for GC-TOF and lipidome metabolites. Paired T-tests were done to calculate significance. 53 mother samples and 36 infant saliva samples and 15 samples from both mother and infant urine samples were used in this analysis.
